# Strengthening stakeholder engagement through ethics review in biomedical HIV prevention trials: opportunities and complexities

**DOI:** 10.1002/jia2.25172

**Published:** 2018-10-18

**Authors:** Catherine Slack, Abigail Wilkinson, Jessica Salzwedel, Paul Ndebele

**Affiliations:** ^1^ HIV AIDS Vaccines Ethics Group (HAVEG) School of Applied Human Sciences College of Humanities University of KwaZulu‐Natal KwaZulu‐Natal South Africa; ^2^ AIDS Vaccine Advocacy Coalition (AVAC) New York NY USA; ^3^ Medical Research Council of Zimbabwe (MRCZ) Causeway, Harare Zimbabwe

**Keywords:** Stakeholder engagement, community engagement, ethics review, Research Ethics Committee, Institutional Review Boards, HIV prevention trials

## Abstract

**Introduction:**

Clinical trials of biomedical HIV prevention modalities require the cooperation of multiple stakeholders. Key stakeholders, such as community members, may have stark vulnerabilities. Consequently, calls for HIV prevention researchers to implement “stakeholder engagement” are increasingly common. Such engagement is held to benefit inter‐stakeholder relations, stakeholders themselves and the research itself. The ethics review process presents a unique opportunity to strengthen stakeholder engagement practices in HIV prevention trials. However, this is not necessarily straightforward. In this article, we consider several complexities. First, is stakeholder engagement a legitimate component of what Research Ethics Committees (RECs) should review for HIV prevention trials? Second, what are the core features of engagement that should be under ethics review? Third, what are the key practices that should be highlighted in ethics review?

**Methods:**

To address these questions, we examined the international ethics guidelines specialized for such trials (UNAIDS 2012, UNAIDS‐AVAC GPP 2011) and directly applicable to such trials (CIOMS 2016; WHO 2011). Thematic analysis was used to code and analyse these guidelines.

**Results and discussion:**

Ethics guidelines support REC review of engagement. Guidance recommends that engagement be broad and inclusive; early and sustained; and dynamic and responsive. Broad engagement practices include evaluating the context, planning in writing, and resourcing. RECs should assess engagement as part of a comprehensive review, and recommend revisions where necessary. Researchers should profile key elements of engagement valued in ethics guidance, when they draft ethics submissions. Importantly, the ethics review process should not undermine the ‘dynamic responsiveness’ required for excellent engagement in this field.

**Conclusions:**

As evidence‐informed engagement strategies emerge, these should inform the ethics submission and review process. Both parties in the review process should strive to avoid a superficial, check‐list type approach that caricatures what should be a thorough, nuanced ethics review of a rich, responsive engagement process.

## 1 Introduction

HIV prevention trials are complex endeavours. Ethics guidelines recognize several background complexities with such trials that trigger the need for stakeholder engagement. UNAIDS 1 notes the pragmatic need for “collaboration” between multiple role‐players for trial success, for example, affected populations, research institutions, industry, government and health sectors (p. 11). Also, there is the vulnerability of key stakeholders, such as participants and community stakeholders, who are at increased risk of potential harm because of marginalization or HIV stigma and discrimination 1. They might be exploited because of disparities in wealth, scientific experience, power, and technical capacity relative to researchers 1. Even host countries are identified as at risk of potential exploitation because of such disparities. Ethics guidelines recognize that when sponsors and researchers engage relevant stakeholders, potential risks and harms can be mitigated.

Ethics guidelines recognize several potential benefits of engagement. *First*, there are beneficial outcomes of engagement for *inter‐stakeholder relations* – that is, relations between researchers and stakeholders that are more trusting, “collaborative,” involve “partnership,” are “mutually beneficial” and “equitable” so that power imbalances are reduced 2. *Second*, there are several beneficial outcomes *for stakeholders themselves* – these include improved knowledge, understanding or literacy; increased trust in research(ers); increased ownership of research 2; and increased acceptance of research 1. *Third*, UNAIDS‐AVAC GPP 2 states there are beneficial outcomes of engagement *for research* – that is, research that is “shap[ed]… collectively” (p.16); that has received effective and expert contributions from stakeholders 2; that is relevant 1; that is culturally appropriate, successfully conducted, where the likelihood of application is enhanced and where a firmer foundation for future research has been laid 2.

Finally, UNAIDS 1 recognizes the “right” of key stakeholders, such as communities, to participate fully as partners in the research, and to make decisions about the nature of their participation (p. 21). Even host countries are identified as having such rights. This rationale is rooted less in the positive consequences of engagement (i.e. reducing risk and enhancing benefits) and more in the inherent “rightness” of involving stakeholders.

The ethics review process presents a unique opportunity to strengthen stakeholder engagement in such trials. Despite rich scholarship on stakeholder engagement 3, 4, 5, 6, 7, 8 little has been written on this specific issue. Ethics review of engagement is not necessarily straightforward. One could question whether review of engagement in HIV prevention trials falls within a Research Ethics Committees’ (RECs) “purview” of responsibility 9. Also, one could question whether any consensus exists about the core features and practices of “excellent” engagement in such trials, such that researchers could highlight these, or RECs could look for these, in the ethics review process.

In this article, we consider several complex questions about leveraging the ethics review process to impact on stakeholder engagement in biomedical HIV prevention trials. *First*, is stakeholder engagement really a legitimate component of what RECs should review?; *second*, what core engagement features should be under ethics review?; and *third*, what core engagement practices should be under ethics review? Given that ethics guidance is central to determining the acceptability of researchers’ practices and of RECs’ practices, we looked to ethics guidance to address these questions.

## 2 Methods

We aimed to find ethics guidelines that would be relevant to any researcher or REC involved in HIV prevention trials anywhere in the world, regardless of host country, institutional affiliation or network membership. We conducted a Google search using a combination of the following key terms – *ethics guidelines* OR *ethics guidance* AND *community* OR *stakeholder* AND *engagement* OR *consultation* OR *participatory* OR *consultation* OR *partnership* OR *involvement* OR *collaboration* AND *biomedical HIV prevention trials* OR *HIV vaccine trials* OR *HIV prevention trials* AND *research ethics committees* OR *ethics review* OR *ethics review committee* OR *institutional review board*.

We included those ethics guidelines *specialized for* HIV prevention trials and those *applicable to* HIV prevention trials conducted internationally. That is, we included UNAIDS (2012) *Ethical Considerations In Biomedical HIV Prevention Trials* which provide guidance on all ethical aspects of such trials 1. We also included UNAIDS‐AVAC GPP (2011) *Good Participatory Practice Guidelines For Biomedical HIV Prevention Trials* 2 which provide guidance on engagement in such trials. We also included the CIOMS (2016) *International Ethical Guidelines for Health‐Related Research Involving Humans* 10 which provide guidance relevant to HIV prevention trials, as a subset of health research with humans. Lastly, we included WHO (2011) *Standards and Operational Guidance For Ethics Review Of Health‐Related Research With Human Participants* 11. We excluded ethics guidelines applicable to specific nations (e.g. South African MRC 2003) 12 or to specific networks (e.g. HPTN 2009)^13^ or non‐HIV diseases (e.g. GPP EP 2016; GPP TB‐Vax 2017; GPP TB‐drug 2012) 14, 15, 16.

Guided by Braun and Clarke's 17 process for Thematic Analysis, each ethics guideline was closely read and coded by two coders, guided by the questions above. For example, for question 2, text we coded as “*early*” included “*at the outset”* and “*at the earliest opportunity”*. Text we coded as “*sustained*” included “*ongoing*” or “*long‐term*.” We clustered codes that had shared meaning to form “sub‐themes” (“*early and sustained*”). We clustered sub‐themes (“*early and sustained*,” “*broad and inclusive*” and “*dynamic and responsive*” into major themes (“features” of sound engagement). We defined qualitative characteristics of engaged research as “features”; and we defined observable conduct or behaviour as “practices.” Discrepancies between coders were resolved by discussion 18. We conducted the search and review during the period June 2017 to May 2018.

## 3 Results

### 3.1 Is “stakeholder engagement” a legitimate part of what RECs should review?

RECs should evaluate engagement when HIV prevention trials are ethically reviewed, according to all ethics guidelines reviewed here. UNAIDS 1 states that ethics review should consider “community participation and involvement” (p. 24). CIOMS 10 states that RECs should receive a “description of the plan for community engagement” (p. 25) (community equates to stakeholder in both cases). WHO 11 states that REC ethics review criteria include “community considerations” (p.14). UNAIDS‐AVAC GPP 2 allow that RECs *can* require the ethics document to be followed, in line with this documents’ tendency to avoid prescriptive language when making recommendations. (It is worth noting but not central to our review that, increasingly, national guidelines also recommend that engagement be reviewed by RECs 19, 20, 21).

### 3.2 What core engagement features should be under ethics review?

Our review identified three broad features. See Table 1.

**Table 1:** Key features of engagement in ethics guidance

Broad and inclusive	
CIOMS (2016)	“Stakeholders are individuals, groups, organizations, government bodies, or any others who can influence or are affected by the conduct or outcome of the research project. The process must be fully collaborative and transparent, involving a wide variety of participants, including patients and consumer organizations, community leaders and representatives, relevant NGOs and advocacy groups, regulatory authorities, government agencies and community advisory boards” (p. 25)
UNAIDS‐AVAC GPP (2011)	“any individual or collection of individuals who have a stake in a biomedical HIV prevention trial” (p. 14)
UNAIDS (2012)	“the concept [of community] needs to be broadened to civil society so as to include advocates, media, human rights organizations, national institutions and governments, as well as researchers and community representatives from the trial site” (p. 18)
**Early and sustained**	
CIOMS (2016)	“Researchers, sponsors, health authorities and relevant institutions should engage potential participants and communities in a meaningful participatory process that involves them in an early and sustained manner in the design, development, implementation, design of the informed consent process and monitoring of research, and in the dissemination of its results” (p. 25)
WHO (2011)	“Researchers should actively engage with communities in decision‐making about the design and conduct of research” (p. 15)
UNAIDS‐AVAC GPP (2011)	“activities required for the development, planning, implementation, and conclusion of a trial, including dissemination of trial results” (p. 5), “a long‐term process that extends throughout and beyond the life‐cycle of any single clinical trial” (p. 66)
UNAIDS (2012)	“engage in consultations with communities who will participate in the trial from the beginning of the research concept, in an open, iterative, collaborative process” (p. 17)
**Responsive and dynamic**	
CIOMS (2016)	“In the design and conduct of the research[…] the researchers and the sponsors must be responsive to the concerns of the community” (p. 63)
UNAIDS‐AVAC GPP (2011)	“The application of each practice or set of practices will vary by location, the type of trial being conducted, and trial site experience” (p. 26), “stakeholders interests, priorities, perspectives, and culture may change over time” (p.16), “Stakeholder identification and inclusion considers the dynamic stakeholder landscape” (p. 31)
UNAIDS (2012)	“engage in consultations with communities […] in an open, iterative, collaborative process” (p. 17), “find solutions to unexpected issues that may emerge once the trial is underway” (p. 17), “Defining the relevant community for consultation and partnership is a complex and evolving process” (p. 18)

#### 3.2.1 Broad and inclusive

Engagement in HIV prevention trials should involve a broad range of diverse role‐players, according to most ethics guidance reviewed here. CIOMS 10 recommends engaging those who can “influence or are affected by” the study (p. 25). UNAIDS‐AVAC GPP 2 similarly recommends engaging those who “have a stake” (p. 14). UNAIDS 1 recommends a broad definition of community. This means engagement should extend beyond community stakeholders who reside locally and represent the interests of participants 2.

#### 3.2.2 Early and sustained

Engagement in such trials should be prompt and continuous, according to all ethics guidelines reviewed here. UNAIDS‐AVAC GPP 2 recommends that it be early and long‐term. CIOMS 10 recommends engagement from design through to dissemination. UNAIDS 1 takes a similar approach. By recommending engagement for “design”, WHO 11 implies early engagement (p.15).

#### 3.2.3 Dynamic and responsive

Engagement in such trials should respond to context, and change across time where needed, according to most ethics guidance reviewed here. UNAIDS‐AVAC GPP 2 acknowledges diverse interests and perspectives that may change across time, and recommends diverse strategies and mechanisms that vary accordingly. UNAIDS 1 recommends an “iterative process” of engagement (p. 18). CIOMS 10 recommends responsiveness to stakeholder concerns. See Table 2.

**Table 2:** Examples of engagement strategies/mechanisms from ethics guidance

UNAIDS (2012) & UNAIDS‐AVAC GPP (2011)	Meetings
UNAIDS (2012) & UNAIDS‐AVAC GPP (2011)	Consultations
UNAIDS‐AVAC GPP (2011)	Local events
UNAIDS‐AVAC GPP (2011)	Suggestion boxes
UNAIDS‐AVAC GPP (2011)	Call‐in radio shows
UNAIDS‐AVAC GPP (2011)	Focus group discussions
UNAIDS‐AVAC GPP (2011)	Interviews
CIOMS (2016) & UNAIDS (2012)	A continuing forum
UNAIDS‐AVAC GPP (2011)	A formal Stakeholder Advisory Mechanism (SAM) e.g. Community Advisory Board (CAB)

### 3.3 What core engagement practices should be under ethics review?

Our review identified three broad practices. See Table 3.

**Table 3:** Core practices of engagement from ethics guidance

Evaluating the context	
CIOMS (2016)	“Active community involvement […] helps the research team to understand and appreciate the research context” (p.5)
UNAIDS‐AVAC GPP (2011)	“Successful stakeholder engagement requires a broad, inclusive, and multifaceted understanding of the context in which a biomedical HIV prevention trial is conducted” (p.16) “Formative research activities can be conducted informally to gather information about local populations and research areas or formally as a part of approved, funded protocols” (p.27)
UNAIDS (2012)	“A social and political analysis should be carried out early on in planning the research process, to assess determinants of vulnerability, such as poverty, gender, age, ethnicity, sexuality, health, employment, education, and legal conditions in potential participating communities”(p.32)
WHO (2011)	“Researchers should actively engage with communities […] while being sensitive to and respecting the communities’ cultural, traditional and religious practices” (p.15)
**Planning in writing**	
CIOMS (2016)	“The research protocol or other documents submitted to the research ethics committee should include a description of the plan for community engagement, and identify resources allocated for the proposed activities. This documentation must specify what has been and will be done, when and by whom” (p.25)
UNAIDS‐AVAC GPP (2011)	“A comprehensive stakeholder engagement plan enables research teams to collaborate with stakeholders and facilitate a more participatory approach to biomedical HIV prevention research” (p. 35)
UNAIDS (2012)	“Scientific and ethical review prior to approval of a trial protocol should take into consideration these issues […] community participation and involvement” (p. 24)
WHO (2011)	“Duties to respect and protect communities require examining by the REC” (p.14)
**Resourcing engagement**	
CIOMS (2016)	“The research protocol or documents sent to the research ethics committee should […] present resources allocated for the community engagement activities” (p.102)
UNAIDS‐AVAC GPP (2011)	“Trial sponsors ensure sufficient funding and research teams allocate resources and time to support stakeholder engagement” (p.45), “Research teams designate trial site staff responsible for [engagement]” (p. 28)
UNAIDS (2012)	“The principal investigator and site research staff should work with representatives of affected communities to identify needs related to their participation, including logistical requirements such as transportation to the meeting site” (p.19)

#### 3.3.1 Evaluating the context

Researchers should understand the context for an HIV prevention trial, according to most ethics guidelines reviewed here. UNAIDS‐AVAC GPP 2 recommends a “multifaceted” understanding (p.16) largely through formative evaluation. CIOMS 10 recommends that engagement “appreciate the research context” (p.5). UNAIDS 1 recommends a socio‐political analysis of background factors. WHO 11 recommends sensitivity to cultural and traditional practices. Such understanding can be achieved informally or formally through dedicated protocols 2, and the latter may require ethics review 2, 10. Researchers should demonstrate in ethics applications to RECs a commitment to evaluating the context so such understanding will be achieved.

#### 3.3.2 Planning in writing

Engagement should be carefully planned and purposeful, according to most guidelines reviewed here. UNAIDS‐AVAC GPP 2 repeatedly recommends planning for engagement. CIOMS 10 and UNAIDS 1 and WHO 11 explicitly recommend that Research Ethics Committees consider engagement, which endorses the requirement for written planning. Plans should include various engagement strategies and mechanisms, *depending on the trial and its context*. See Table 2 for a non‐exhaustive, non‐prescriptive list. These strategies can elicit concerns, objections, advice, experiences, expectations, needs, preferences, perceptions, perspectives, beliefs, inputs, feedback, responses, recommendations and suggestions and other crucial information relevant to trials 1, 2, 10. Researchers should demonstrate in ethics applications to RECs that their engagement is carefully planned.

#### 3.3.3 Resourcing

Engagement should be sufficiently resourced, according to most ethics guidelines reviewed here. UNAIDS‐AVAC GPP 2 strongly endorses funding and staffing for engagement, in no less than 15 places, spanning site selection to post‐trial access. CIOMS 10 recommends that resources allocated for engagement be declared to RECs. UNAIDS 1 simply recommends that logistics for consultations be addressed. Researchers should demonstrate in ethics applications that they have carefully considered the issue of resources for engagement.

## 4 Discussion

RECs should review engagement because ethics guidelines governing or applicable to HIV prevention trials explicitly assign this responsibility to RECs. Both researchers and RECs should understand this. However, in order to deliver “well‐reasoned judgements” (p. 457) 22 and avoid “poorly justified responses” or “unjustified variations” 23 (p.15) in judgements we hope that RECs will use norms in guidance to render their judgements (more below), while not undermining “efficient processes” 22, 23. We also argue that review of engagement is supported by a leading ethics framework, namely, the “Emanuel Framework” 24, 25. The Emanuel Framework 24 provides a comprehensive and coherent way for “ethics reviewers” to “evaluate a protocol and to determine whether it fulfils ethical standards” (p. 131‐132). It explicitly provides ethics reviewers with an organized way to conceptualize “what [they] already do” (p. 132). It positions stakeholder engagement (termed “collaborative partnership”) as the first component of ethical research 5, thereby firmly situating it as part of a comprehensive ethics review. “Collaborative partnership” recognizes community stakeholders and policy‐makers as critical stakeholders for consultation, in order to fully realize the potential benefits of research.

Researchers should state their engagement plans to RECs in a way that facilitates review of key elements valued in ethics guidance (more below). They may also need to assess national ethics guidelines to see if unique, additional local recommendations exist for researchers and sponsors. Certain RECs, or members, may not necessarily have the expertise in stakeholder engagement, and may find this paper helpful in crystallizing core ethics recommendations from ethics guidance. They should use ethics norms to evaluate whether the planned engagement is acceptable. More specifically, ‘*broad and inclusive*’ means RECs can assess whether engagement appears overly focussed on any one stakeholder (e.g. CABs) and inquire about other stakeholders, where necessary. This broadened understanding resonates with key literature 5, 6. CABs may provide a formal mechanism for soliciting the views of various community stakeholders, as well their expertise 26. However, ethics submissions should describe engagement that is “beyond CABs.” Ethics submissions could describe sponsor or network efforts to date to engage international and national stakeholders (or “multiple‐level” engagement) 27.

‘*Early and sustained*’ means RECs can assess whether engagement appears overly focussed on any one stage (e.g. recruitment or results‐dissemination). Post‐recruitment engagement has been the focus of recent scholarship 8. “Dynamic responsiveness” means RECs can evaluate whether engagement practices are appropriately tailored to the study and context, and whether plans can accommodate unexpected issues. Several commentators have recommended that engagement strategies be tailored and flexible 8, be improved via constant feedback 4, be revised in response to unfolding issues and realities 6 and take into account the dynamic, transient nature of various groups 28.

In terms of key practices, RECs can address ‘*evaluation of the context*’ by helping researchers to judge whether evaluation activities constitute “formal research,” and where they do, ethics reviewers could evaluate whether such activities meet norms for research, such as social value or scientific validity 24. The importance of researcher understanding of the socio‐economic‐political context has been strongly endorsed 5. Seeing ‘*planning*’ as a core practice means RECs should seek evidence of this in the ethics submission. RECs should recognize that ethics guidelines do not take a firm stand on which ethics submission document should outline engagement plans nor in what detail 10. RECs should recognize that sufficient information is needed for them to assess whether ethics norms are met, while preserving researchers’ needs for responsiveness 29. In order to satisfy the ‘*resourcing*’ aspect, RECs should recognize various ways to satisfy this ‐ e.g. declarations by the applicant that engagement is funded; or review of the actual budget. Because funding for engagement might detract from funding for other aspects (e.g. data collection) researchers should follow a transparent, fair process in budget allocation and RECs should ensure that an appropriate balance has been struck.

Researchers and RECs should not inadvertently undermine ‘*dynamic and responsive*’ engagement through their actions in the review process. Researchers should describe plans to RECs in a way that preserves nimble future responses, and be forthcoming that their engagement plans will, and should, be adjusted in an attuned manner to a dynamic context. Various commentators such as Tindana et al. 30 recommend that engagement is flexible to “meet changing needs” (p. 1453), and MacQueen et al. 5 recommend “a dynamic process that is imbued with feedback loops” (p. 7). RECs should not require amendments to be submitted (as they might for trial procedures), because they should promote rapid engagement responses to future unforeseen developments. In certain instances, RECs may wish to be notified of new engagement efforts, for example, where the rights and welfare of community or other stakeholders are substantially affected, and where additional ethics paperwork is justified. RECs should not routinely insist on submission of granular operational ‘living documents’ best left to research sites, such as CAB membership lists. Both parties should draw on analogies with the review of consent processes – where RECs assess broad plans for consent strategies (e.g. regular review of consent concepts) while enabling researchers to implement consent practices that respond to the needs of individual participants in context.

Ideally, when RECs judge that planned engagement does not meet ethics recommendations, they should not recommend rejection of a protocol but rather make constructive recommendations for improvement so plans resonate better with ethics guidance. RECs can try to recruit persons with such expertise, or consult such experts as *ad hoc* reviewers if need be, or utilize the expertise of community members. Also, key REC documents should accommodate review of engagement. For example, application forms for initial review should have questions that will “trigger” researchers to describe their engagement practices in a way that is ‘*broad and inclusive*’ (e.g. more than permission from “institutional gatekeepers”) 31. Renewal forms (progress reports) should enable researchers to describe progress in the preceding year, to promote ‘*sustained*’ engagement. This might impact the percentage of inquiries that RECs raise about engagement 32, 33, 34.

Ethical responses evolve over time, requiring researchers and RECs to stay abreast of concerns affecting their responsibilities. Both might benefit from training that highlights engagement as a legitimate focus of *ethics review* 35. This might complement existing research ethics modules 36, 37, 38, 39, 40, 41 developed by several institutions 42, 43, 44, 45, 46, 47, 48 that highlight practical skills using interactive features 36, 39, 41, 49, 50, 51, 52, 53, 54, 55, 56. This might also complement existing modules featuring engagement as a key part of ethical research (FHI 360) 46; of ethical HIV vaccine trials, of ethical adolescent trials, and of public health research (TRREE) 42.

Ideally, evidence‐based “best practices” for engagement should inform ethics submissions, as data become available, including for monitoring such practices. It is recognized that more evidence is needed for the impact of engagement on key outcomes, and several studies report on perceived impact 57, 58, 59, 60. There is also renewed commitment to building an evidence base 61. Ethics guidelines, however, are clear that engagement holds potential benefit for inter‐stakeholder relations, stakeholders themselves and research itself. This issue may have an historical parallel in the consent arena, where ethics guidance called for participant understanding before evidence existed about effective strategies 62.

Our review has several limitations. First, by limiting ourselves to cross‐nation, cross‐network, cross‐institution ethics guidance, several features and practices relevant to ethics review of engagement under specific circumstances may have been excluded, for example monitoring and evaluation. Also, because our coding was driven by our specific questions related to ethics review of engagement, it is possible that alternate questions may have yielded new or additional codes 17.

## 5 Conclusions

Ethics review of HIV prevention trials affords researchers and RECs an opportunity to highlight core elements of engagement valued in ethics guidance. We found that ethics guidance recommends that engagement for such trials be broad, sustained, responsive, based on nuanced understanding of the context, carefully planned, and importantly be adequately resourced. Both parties in the review process should strive to avoid a superficial, check‐list type approach 6 that caricatures what should be a nuanced, sensitive ethics review of a rich, reflexive engagement process.

## Competing Interest

The authors declare that they have no competing interests.

## Authors contributions

CS designed the paper, reviewed ethics guidelines and drafted the manuscript; AW reviewed ethics guidelines and drafted the manuscript; JS helped interpret the review and revised the manuscript for important content; PN helped interpret the review and revised the manuscript for important content.
